# Multiparametric magnetic resonance imaging findings of prostatic pure leiomyomas

**DOI:** 10.1590/S1679-45082016AO3715

**Published:** 2016

**Authors:** Thais Caldara Mussi, Yves Bohrer Costa, Marcos Takeo Obara, Marcos Roberto Gomes de Queiroz, Rodrigo Gobbo Garcia, José Antonio Domingos Cianciarulo Longo, Gustavo Caserta Lemos, Ronaldo Hueb Baroni

**Affiliations:** 1 Hospital Israelita Albert Einstein, São Paulo, SP, Brazil.

**Keywords:** Prostate, Leiomyoma, Magnetic resonance imaging

## Abstract

**Objective:**

To describe the imaging findings of prostatic tumors nonadenocarcinoma on multiparametric magnetic resonance imaging.

**Methods:**

A total of 200 patients underwented multiparametric magnetic resonance imaging of the prostate for screening for prostate cancer, from August 2013 to September 2014, followed by biopsy with ultrasound/magnetic resonance imaging fusion.

**Results:**

We found three pathologic proved cases of prostatic pure leiomyomas (0.02%) in our series and described the multiparametric magnetic resonance imaging features of these prostatic leiomyomas. The imaging findings had similar features to lesions with moderate or high suspicion for significant cancer (Likert 4 or 5) when localized both in the transitional zone or in the peripheral zone of the gland.

**Conclusion:**

Pure prostatic leiomyomas had imaging findings on multiparametric magnetic resonance imaging that mimicked usual adenocarcinomas on this test. Radiologists, urologists and pathologists must be aware of this entity and its imaging features.

## INTRODUCTION

Pure leiomyoma of the prostate is a rare entity with origin in the Mullerian duct remnant, the prostatic capsule or the periglandular prostatic tissue.^(1)^ Until 1951, the definition of prostatic leiomyoma was confuse, and Kaufman et al. arbitrarily described it as “a circumscribed or encapsulated mass of smooth muscle, measuring 1cm or longer in diameter, containing varying amounts of fibrous tissue, but devoid of glandular elements, and which is either obviously prostatic or juxta-prostatic, in origin and position”. Their aim was to distinguish it from fibromuscular hyperplasia, bladder neck tumors or clinically insignificant nodules that created discrepancies in the literature.^(2)^ The differential diagnosis between leiomyomas (rare) and stromal nodules (more common) is difficult and sometimes not possible, but differentiation is based on the fact that leiomyomas have well-organized fascicles.^(3,4)^

There are less than 100 cases of pure prostatic leiomyomas described in the literature, and in almost all cases the tumor was large and the patient presented with urinary and anorectal symptoms, or an altered digital rectal examination.^(1,2,5)^ There are few case reports in the literature describing large prostatic leiomyomas on magnetic resonance imaging (MRI).^(1,6,7)^ However, to our knowledge, there is no article describing the imaging findings of pure prostatic leiomyomas in asymptomatic patients submitted to multiparametric MRI (mpMRI) of the prostate.

## OBJECTIVE

To describe the imaging findings of prostatic leiomyomas on multiparametric magnetic resonance imaging.

## METHODS

### Patient population

From August 2013 to September 2014, a total of 200 patients were submitted to mpMRI before biopsy, in a period up to 6 months. Of those, 189 had an MRI for prostate cancer screening, five for staging and six were in active surveillance. The study was approved by the Research Ethics Committee of the *Hospital Israelita Albert Einstein* (HIAE), under protocol number 1.446.587, CAAE: 53632916.1.0000.0071.

### Magnetic resonance imaging technique and interpretation

All patients underwent mpMRI of the prostate on a 3 Tesla system (Magnetom Trio, Siemens HealthCare, Erlangen, Germany) using a pelvic phased-array coil. Pre-contrast sequences of the prostate and seminal vesicles included high resolution axial, coronal, and sagittal turbo-spin echo (TSE) T2-weighted images (WI), axial TSE-T1-WI, and axial fat-suppressed single-shot echo-planar diffusion-weighted images (DWI). Diffusion-weighted images were obtained with b values of 50, 400 and 800, with reconstruction of apparent diffusion coefficient maps. In addition, dynamic contrast-enhanced imaging of the prostate and seminal vesicles was obtained using an axial 3D fat-suppressed T1-WI spoiled gradient-echo sequence obtained before and ten times after intravenous administration of 0.1mmol/kg of gadopentetate dimeglumine (Magnevist, Bayer Heatlhcare Phamarceuticals, Whippany, NJ, USA), with a 12-second temporal resolution.

All mpMRI cases were graded using a 5-point scale, according to the probability of having a clinically significant prostate cancer, ranging from highly unlikely to highly likely to be present.

### Biopsy technique

As routinely performed in our institution, all patients underwent ultrasound/magnetic resonance imaging fusion for prostate biopsies, with systematic sampling of 12 regions of the peripheral zone, and 2 of the central gland, and additional sampling of the suspicious areas, if present. One of two different ultrasound pieces of equipment was used to fuse the images and perform the biopsies: Aplio 500 with Smart Fusion (Toshiba Medical System Corporation, Minato, Tokyo, Japan) or LOGIC E9 with imaging fusion software (GE Healthcare, Little Chalfont, United Kingdom). We obtained adequate pathological proof of the results through biopsy samples in all these patients.

## RESULTS

**Figure 1:**
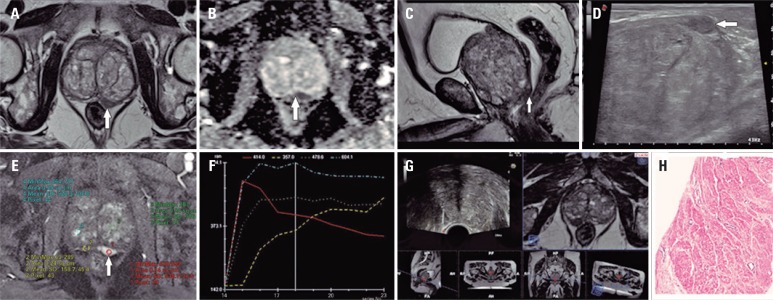
A 73-year-old man with prostate specific antigen level of 6.6ng/mL, palpable nodule on digital rectal examination and negative prior biopsy. Multiparametric magnetic resonance imaging of the prostate shows a well-defined nodule, with very low signal intensity in the peripheral zone on T2-WI (A and C), intense restricted diffusion demonstrated by a low signal on apparent diffusion coefficient map (B) and hypervascularized pattern, with an early wash-in followed by wash-out curve (E and F). Real time imaging fusion ultrasound/magnetic resonance imaging technique: the nodule seen as a low-signal lesion in the peripheral zone of the prostate on T2-WI (C) appears as a round hypoechoic nodule on ultrasound (D). Systematic biopsy with additional samples of the suspicious area was performed (note that magnetic resonance imaging images were inverted during the procedure) (G), and pathological results demonstrated a pure prostatic leiomyoma (H)

**Figure 2:**
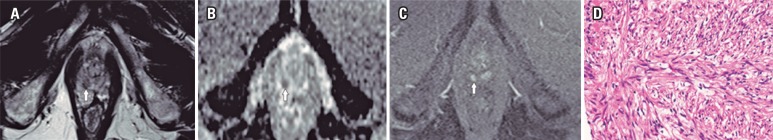
Multiparametric magnetic resonance imaging of the prostate shows a circumscribed nodule in the peripheral zone on T2-WI (A), with moderate restricted diffusion on the apparent diffusion coefficient map (B) and early hypervascularization on the dynamic contrast-enhanced sequence (C). Pathological analysis confirmed a pure prostatic leiomyoma (D)

**Figure 3:**
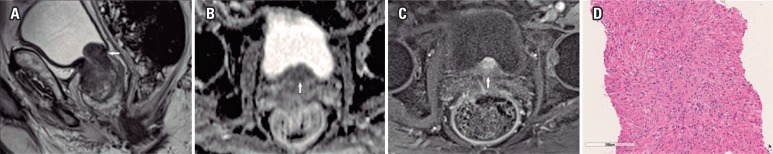
Multiparametric magnetic resonance imaging of the prostate shows a poorly-defined lesion in the periurethral area of the transitional zone (A), with moderate restricted diffusion on the apparent diffusion coefficient map (B), and early hypervascularization on the dynamic contrast-enhanced sequence (C). Pathological analysis confirmed a pure prostatic leiomyoma (D)

From the 200 biopsies, 83 (41.5%) were negative and 117 (58.5%) positive for neoplasia. Of those 117 positive cases, 113 (96.6%) were acinar adenocarcinomas, 1 (0.008%) was a stromal tumor of uncertain malignant potential and 3 (0.02%) were leiomyomas. The clinical and imaging characteristics of the three leiomyomas are described below, and figures 1 to 3 illustrate the imaging and pathological findings of these cases.

### Imaging findings

The imaging findings of these three leiomyomas had moderate to high suspicion for clinically significant prostate cancer on mpMRI (Likert 4 and 5).

Case number 1 was a 73-year-old man with prostate specific antigen (PSA) of 6.6ng/mL, palpable nodule on digital rectal examination and negative prior biopsy. mpMRI showed a 12mm round nodule in the left mid-gland of the peripheral zone that abuts the prostate contour, with very low signal on T2-WI, marked restriction diffusion and early enhancement with washout. It was classified as clinically significant disease highly likely to be present (Likert 5) (Figure 1).

Case number 2 was a 60-year man with PSA of 1.7ng/mL and alteration in the right apex upon digital rectal examination. mpMRI showed a 8mm round nodule in the right apex of peripheral zone, with low signal on T2-WI, moderate restriction diffusion and enhancement. It was classified as clinically significant disease likely to be present (Likert 4) (Figure 2).

Case number 3 was an 81-year-old man with PSA level of 7.0ng/mL in active surveillance and normal digital rectal examination. mpMRI showed a 28mm poorly-defined lesion, in the periurethral area of the transitional zone, with low signal on T2-WI, restriction diffusion and early enhancement. It was classified as clinically significant disease likely to be present (Likert 4) (Figure 3).

All cases were readily detected on ultrasound during the fusion biopsy as well-circumscribed, hypoechoic lesions.

### Pathological findings

Slides showed fragments of biopsy composed of spindle smooth muscle cells, separated by small amounts of collagen. The tumor cells were arranged in an orderly pattern of intersecting fascicles. Individual cells had blunt-ended nuclei with evenly distributed nuclear chromatin. There was no nuclear atypia, tumor necrosis or mitotic activity. Tumor cells had positive immunostaining for desmin and smooth muscle actin. Epithelial elements were not seen within the nodules.

## DISCUSSION

The use of prostate mpMRI prior to biopsy is well known and established in the literature to increase the accuracy of the procedure.^(8–12)^ The positivity of clinically significant prostate cancer in biopsy is directly related to suspicion grade on mpMRI, and in moderate to highly suspicious cases the positivity of the biopsy ranges from 73 to 100%.^(8,13)^

We described three cases of pure prostatic leiomyoma on biopsies with ultrasound/MRI imaging fusion, all with a likely to highly likely probability of clinically significant prostate cancer on mpMRI using a probability classification.

The etiology of prostatic leiomyoma remains unknown and some authors believe that has embryological anlagen from Mullerian remnants. Other hypotheses are that inflammation and infection transform glandular tissue in smooth muscle and the hypertrophy of this muscle produces myoma, or that infection and arteriosclerosis degenerate the hyperplastic tubular nodules, remaining just smooth muscle stroma and scar tissue.^(4,6,14)^ Most cases are diagnosed as large lesions or in patients presenting with urinary symptoms (obstruction or infection).^(6)^ To our knowledge, there is no study describing the imaging findings of asymptomatic prostatic leiomyomas.

Treatment for prostatic leiomyomas is controversial. Although large lesions are usually surgically removed through radical prostatectomy, smaller tumors, such as in our report, remain a challenge for the urologist (given the possibility of growth, recurrence and malignant transformation, even being rare). Hence, therapeutic decisions should be individualized.^(15)^

## CONCLUSION

Prostatic leiomyomas are lesions that have imaging findings on multiparametric magnetic resonance imaging suspicious for clinically significant prostate cancer. They may present as important mimickers of adenocarcinomas in both peripheral zone and transitional zone of the prostate on multiparametric magnetic resonance imaging. With the increasing use of multiparametric magnetic resonance imaging as a screening tool before prostate biopsy, it is likely that diagnosis of leiomyoma will increase; thus radiologists, urologists and pathologists must be aware of this entity.
